# Molecular Determinants of IgE-Mediated Food Allergenicity: Structural Dynamics, Processing-Induced Remodeling, and Analytical Interpretation

**DOI:** 10.3390/molecules31213950

**Published:** 2026-11-02

**Authors:** Hejun Duan, Yixing Feng, Xin Zhang, Yumin Niu

**Affiliations:** Beijing Key Laboratory of Diagnostic and Traceability Technologies for Food Poisoning, Beijing Center for Disease Prevention and Control, Beijing 100013, China; dhj@vip.163.com (H.D.); fengyxioz@126.com (Y.F.); f_zhangxin@163.com (X.Z.)

**Keywords:** IgE-mediated food allergy, molecular architecture, epitope accessibility, food processing, food matrix, gastrointestinal digestion, allergen detection, exposure assessment, risk interpretation

## Abstract

Immunoglobulin E (IgE)-mediated food allergenicity is not determined by protein identity alone. Processing, food matrix interactions, and gastrointestinal transit reshape allergen molecular states, while clinical consequences also depend on exposure. These transformations can alter the persistence, epitope accessibility, and digestive susceptibility of allergen-derived species. This narrative review examines how molecular architecture shapes these changes and their consequences for IgE binding, effector-cell activity, and analytical detectability. Examples from major allergen families show why structural stability and digestive persistence must be interpreted in context. Processing may reduce, preserve, or enhance immunoreactivity depending on the allergen, matrix, conditions, and endpoint measured. Persistence of intact proteins or IgE-binding fragments does not, by itself, establish functional activity or clinical elicitation. Analytical interpretation depends on the measurand: methods targeting different molecular features do not provide interchangeable estimates of intact allergen abundance or biological activity. Changes in analytical signals may reflect extraction efficiency, epitope accessibility, or target recovery rather than proportional changes in biologically relevant allergenic material. By connecting molecular transformation with analytical measurement, this review examines how validated analytical results can inform exposure assessment and comparison with reference doses in processed foods, alternative proteins, and complex formulations. Interpretive claims should therefore be aligned with the structural, immunological, analytical, or clinical evidence available.

## 1. Introduction

Food allergy is a major concern for global food safety and public health. However, current prevalence estimates, clinical phenotypes, and diagnostic categories alone cannot explain the molecular behavior of food allergens [1]. Allergic responses arise from immune recognition of food-derived proteins or molecular species that persist through processing and digestion [2,3,4]. The relevant question is not simply whether a source ingredient contains an allergen, but which allergen-derived species are present, accessible, and biologically active under a given combination of food composition, processing history, and host physiology.

This review focuses on immunoglobulin E (IgE)-mediated food allergy. Sensitizing capacity refers to the ability of exposure to induce allergen-specific IgE; IgE binding to antibody recognition of a molecular species; biological activity to functional responses such as basophil or mast-cell activation; and clinical elicitation to symptoms triggered in sensitized individuals at a defined exposure. These endpoints are related but non-interchangeable, and a change in one does not establish a proportional change in another [4,5].

Several terms are used in a restricted sense. Digestive stability denotes resistance to gastrointestinal degradation, whereas digestibility describes the extent or process of degradation. Persistence refers to molecular species remaining after a processing or digestive stage. Bioaccessibility denotes accessibility within the gastrointestinal environment and is distinct from epithelial transport or systemic bioavailability. Antigenicity is used only for broader antibody recognition, whereas “hypoallergenic” is treated as an evidence-dependent descriptor whose meaning depends on the molecular, IgE-binding, functional, or clinical endpoint.

Food allergens are better viewed as dynamic biomolecular systems than as fixed molecular entities. Their structure, solubility, epitope accessibility, protease susceptibility, and analytical recovery may change during processing, interaction with the food matrix, and gastrointestinal transit [2,3,6,7]. Conformational stability, epitope organization, intermolecular association, chemical modification, digestive resistance, and host-specific immune recognition together shape the molecular states that persist along this trajectory. The same allergenic protein can display different IgE-binding profiles, digestion products, and analytical recovery depending on the matrix and processing conditions.

Food processing is a major driver of this remodeling. Its effects on allergen structure and epitope accessibility depend on the allergen, the food matrix, and the processing conditions [6,7,8]. These changes may alter epitope accessibility, solubility, protease access, and analytical recovery and should not be assumed to uniformly reduce allergenicity [8,9,10].

Analytical interpretation depends on the measurand. Different analytical platforms target distinct molecular features, and their outputs are therefore not interchangeable proxies for intact allergen abundance, biological activity, or clinical risk [9,10,11,12]. Changes in analytical signals may reflect altered extraction efficiency, modified epitope accessibility, or changed target-peptide recovery rather than proportional changes in the amount of biologically relevant allergenic material [11,12,13]. Biological interpretation therefore requires analytical measurements to be considered alongside molecular state, IgE recognition, functional evidence and, where available, clinical evidence [9,10].

This is a narrative rather than a systematic review. It synthesizes mechanistic and analytical literature on molecular architecture, processing, food matrix effects, and gastrointestinal transformation. It also addresses analytical measurement, exposure assessment, and threshold-based risk interpretation. Primary studies, reviews, methodological frameworks, and authoritative guidance are used illustratively rather than exhaustively. Clinical presentation, diagnosis, and management of IgE-mediated food allergy have been addressed in a dedicated overview [14]. More recent EAACI guidelines provide separate evidence-based recommendations for the diagnosis [15] and management [16].

OIT illustrates why desensitization and protection against allergic reactions should be assessed separately. It increases the tolerated allergen dose during treatment, but sustained unresponsiveness after cessation is less consistent, and treatment-dose reactions remain an important limitation [16]. In peanut allergy, OIT improves supervised food-challenge outcomes but is associated with more allergic and anaphylactic reactions than allergen avoidance or placebo [17]. This contrast demonstrates that improvement on one endpoint does not necessarily translate into corresponding improvement on another. The same caution applies when molecular measurements are interpreted in relation to clinical risk.

**Figure 1:**
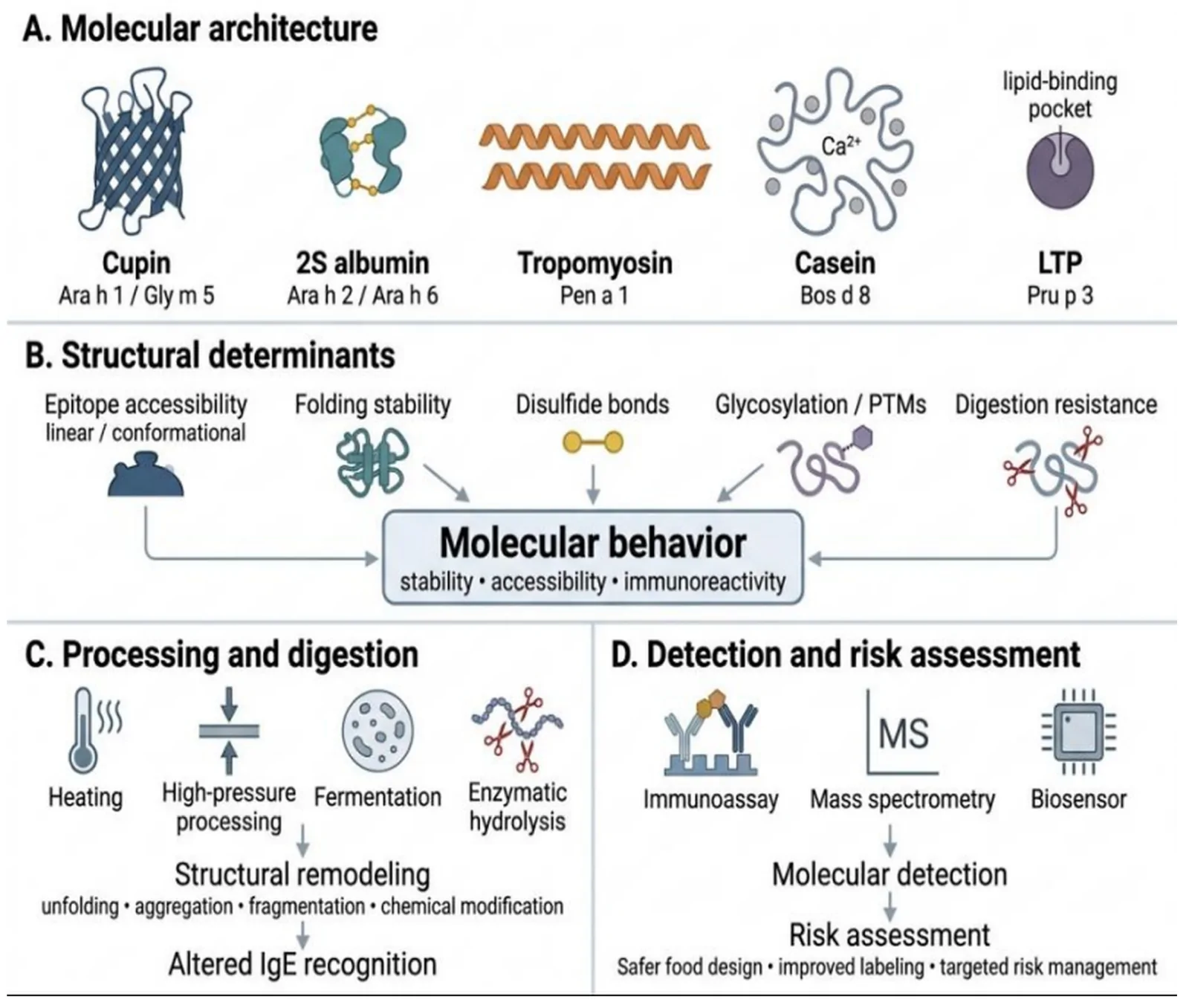
Molecular architecture and dynamic behavior of major food allergens. Representative allergens are grouped within conserved protein families. Their behavior is shaped by epitope accessibility, folding stability, ligand binding, disulfide-bond networks, glycosylation and other post-translational modifications, aggregation, and digestion resistance. These properties influence molecular trajectories through processing, matrix interactions, gastrointestinal transformation, analytical measurement, exposure assessment, and risk interpretation. The relationships are context-dependent and should not be interpreted as deterministic predictors of clinical elicitation. Abbreviations: IgE, immunoglobulin E; LTP, lipid transfer protein; MS, mass spectrometry; PTMs, post-translational modifications.

This review follows allergen-derived molecular species from food to consumer and examines how their molecular state changes during processing, within food matrices, and throughout gastrointestinal transit. It then considers how these changes affect analytical interpretation and how analytical measurements can inform exposure assessment, comparison with reference doses, and risk interpretation. Clinical and epidemiological evidence is included only where it helps contextualize the molecular and analytical findings. Figure 1 summarizes this framework.

## 2. Molecular Architecture of Major Food Allergens

Despite substantial taxonomic diversity, most well-characterized food allergens belong to a limited set of protein families [18,19,20,21,22,23,24]. Their recurrent appearance suggests that particular molecular architectures may favor molecular persistence, cross-recognition, and retention of immunologically accessible surfaces.

The molecular consequences of these architectures are context-dependent: processing, matrix association, and gastrointestinal transformation can remodel allergen molecular states before they reach the intestinal immune interface. Compact, disulfide-rich proteins may retain organized domains after processing or digestion, whereas conformationally labile proteins may lose their native fold while preserving linear IgE-reactive sequences. Aggregation and ligand binding can further expose, mask, or stabilize allergenic determinants [2,3,6,7,18].

No single structural attribute reliably predicts clinical elicitation. Greater stability or resistance to digestion may prolong exposure to immunologically relevant species, but neither property alone determines sensitization, reaction threshold, or severity. Some clinically important allergens are readily digested, whereas many digestion-resistant dietary proteins are not major allergens. The more relevant question is which molecular species persist along the food-to-gut trajectory, in what abundance, and which immune-interacting surfaces remain accessible [2,3,4,5,25].

### 2.1. Structural Features Governing Allergen Molecular Behavior

The behavior of food allergens depends on how molecular architecture constrains conformational changes and surface accessibility. These properties shape their responses to processing and digestion, including changes in epitope exposure and protease susceptibility.

Compact folding can support molecular persistence by limiting solvent penetration and restricting protease access to susceptible peptide bonds. The peanut allergens Ara h 2 and Ara h 6 illustrate this relationship. Both are 2S albumins with compact, disulfide-stabilized structures. Comparative digestion studies have shown that Ara h 2 and Ara h 6 are more resistant to pepsin than the major peanut cupins Ara h 1 and Ara h 3, and that digestion-resistant fragments can retain IgE reactivity [26,27,28]. Selective disruption of disulfide bonds stabilizing the protease-resistant core of Ara h 6 increased susceptibility to tryptic proteolysis and reduced the IgE-binding and mast-cell-activating capacity of the digestion products under the conditions tested [27].

Plant nsLTPs provide another example of covalently constrained architecture. The prototypic peach allergen Pru p 3 adopts a compact four-helix bundle stabilized by four conserved disulfide bonds surrounding an internal hydrophobic cavity [29]. The cherry nsLTP Pru av 3 retains immunological activity more effectively after heating and digestion than the relatively labile PR-10 and profilin allergens from the same fruit [30]. Even within the nsLTP family, ligand occupancy can modify conformational dynamics, thermal stability, proteolytic susceptibility, and IgE-binding capacity. Under some conditions, the allergen and its bound ligand may behave as an integrated molecular unit [31,32,33].

Structural compactness does not universally predict persistence. Caseins, for example, are flexible phosphoproteins that form dynamic micellar assemblies rather than conventional globular structures. Their allergenic behavior is better interpreted through colloidal state, matrix association, and epitope accessibility than compact tertiary folding [18].

PR-10 allergens such as Ara h 8 and Mal d 1 adopt a Bet v 1-like fold and are generally more labile to heating and digestion than many disulfide-rich plant allergens. Their behavior still varies among isoforms and processing conditions, so family membership alone does not predict clinical elicitation [3,34].

Ligand binding further illustrates the context dependence of allergen architecture. Parvalbumins and α-lactalbumin bind calcium, whereas nsLTPs and β-lactoglobulin bind lipids or other hydrophobic ligands. Ligand occupancy can stabilize folding, reorganize conformational epitopes, or alter aggregation and digestion. Glycosylation can likewise reshape the protein surface, alter solubility, and introduce glycan determinants involved in antibody recognition. These effects are protein- and ligand-specific and cannot be inferred reliably from the unliganded structure alone [32,35,36,37].

### 2.2. Major Allergen Protein Families and Conserved Structural Motifs

The cupin superfamily includes 7S vicilins and 11S legumins, exemplified by peanut Ara h 1/Ara h 3 and soybean Gly m 5/Gly m 6. Conserved cupin domains form higher-order oligomers that can reorganize and aggregate during heating; their behavior after processing therefore cannot be inferred solely from the native oligomeric state.

The 2S albumins, represented by Ara h 2, Ara h 6, Ses i 1, and Ber e 1, possess compact α-helical scaffolds stabilized by conserved disulfide-bond networks [19,20,26,27,28]. These covalent constraints can limit complete unfolding and support the persistence of immunoreactive fragments [20,26,27,28]. Their prominence among peanut, sesame, and tree nut allergens is consistent with this structural persistence, although allergen abundance, epitope properties, and host sensitization also influence which members of this family emerge as major allergens.

Plant nsLTPs, including Pru p 3, Ara h 9, and Cor a 8, share a four-helix bundle stabilized by four conserved disulfide bonds and containing a flexible hydrophobic cavity [29,30,31]. This conserved architecture supports structural stability and cross-recognition, although individual nsLTPs differ in surface and epitope properties, ligand occupancy, expression, and biological activity [29,31,32,33].

PR-10 proteins such as Ara h 8, Mal d 1, and Cor a 1 adopt a Bet v 1-like fold and contribute to pollen–food cross-recognition. Their processing and digestion behavior varies among isoforms and experimental conditions [3,34]. Profilins constitute another widely distributed family of plant allergens. Their conserved actin-binding fold contributes to broad IgE cross-recognition among pollen and plant-food homologues, whereas their comparatively limited thermal and digestive stability distinguishes them from more persistent plant allergens such as many nsLTPs and 2S albumins [34,38].

Art v 1-related defensins provide another example of structure-mediated cross-reactivity. Art v 1, the major mugwort pollen allergen, contains a defensin-like domain linked to a proline-rich region, whereas related food defensins include Api g 7 from celeriac. IgE cross-reactivity between Art v 1 and Api g 7 may contribute to some mugwort-associated food allergies, although relatively few food defensins have been characterized and IgE binding alone does not establish clinically relevant cross-allergy [39].

Tropomyosin is a major allergen in crustacean and molluscan foods. Its elongated α-helical coiled-coil structure and high sequence conservation underpin cross-recognition among invertebrate species. However, serological cross-sensitization does not by itself establish a clinically relevant food allergy [24].

Fish parvalbumins are small calcium-binding proteins whose allergenic activity varies among species and isoforms despite broad structural conservation. Calcium occupancy can influence protein conformation and epitope organization, but its effects on epitope accessibility and IgE recognition remain protein- and assay-dependent [35].

Milk contains structurally diverse allergenic proteins. Caseins are flexible phosphoproteins organized in micellar assemblies, whereas β-lactoglobulin and α-lactalbumin are compact whey proteins. These distinct architectures contribute to different responses to processing, with changes in solubility, aggregation, and immunoreactivity depending on the individual protein, matrix, and processing conditions [18,40].

Wheat gluten proteins extend this structural framework. Gliadins are predominantly monomeric, whereas glutenins form disulfide-linked polymers. Among the gliadins, ω-5 gliadin (Tri a 19) is a clinically important allergen in wheat-dependent exercise-induced anaphylaxis [22,41]. Processing modifies gluten-network assembly and protein extractability, complicating interpretation of both molecular behavior and analytical target selection [22,41].

Sesame and soybean further broaden the range of relevant molecular architectures. Sesame contains several characterized allergens, including structurally persistent seed-storage proteins, whereas soybean contains allergens from multiple protein families with distinct structural and processing characteristics [20,21,42]. These differences limit the direct extrapolation of molecular behavior or analytical detectability across seed and legume allergen systems.

Among egg-white allergens, ovomucoid (Gal d 1) is a disulfide-rich Kazal-type protease inhibitor with substantial heat and digestive stability, whereas ovalbumin (Gal d 2), ovotransferrin (Gal d 3), and lysozyme (Gal d 4) belong to distinct structural families. Egg-white allergen behavior therefore reflects the combined properties of structurally distinct proteins rather than a uniform molecular response [23,43].

### 2.3. Linear and Conformational Epitopes

Allergen architecture matters immunologically through the regions recognized by antibodies and T cells. Linear B-cell epitopes contain contiguous residues, whereas conformational epitopes arise from residues brought into spatial proximity by protein folding [44]. Linear IgE-binding sequences may remain recognizable after loss of native tertiary structure if relevant residues remain intact and accessible. Thus, thermal denaturation or proteolysis does not necessarily abolish immunoreactivity. T-cell epitopes are even less dependent on native folding because proteins are processed into peptides before major histocompatibility complex class II presentation.

Conformational epitopes depend more strongly on native or partially native molecular surfaces. Heating, pressure, pH changes, disulfide reduction, or ligand loss can disrupt IgE-recognized spatial arrangements, but this does not necessarily imply reduced biological activity or clinical elicitation. Unfolding may expose buried sequences, while aggregation can stabilize partially ordered structures or increase local IgE-binding density [2,6,7]. Extensive heating in baked matrices likewise has protein-, matrix-, and endpoint-dependent effects. In vitro digestion of baked muffin systems altered egg and peanut allergen bioaccessibility and IgE reactivity differently, which should not be interpreted alone as evidence of clinical tolerance [11].

Epitope presence and accessibility are distinct. An IgE-reactive sequence may remain intact yet become inaccessible after aggregation or association with matrix components, while partial unfolding may expose previously buried sequences. For immunoassays, reduced antibody binding can therefore reflect epitope masking, poor extractability, or loss of assay-antibody recognition rather than destruction of the allergenic protein. Conserved sequences or conformational surfaces within PR-10 proteins, nsLTPs, tropomyosins, and parvalbumins can support IgE recognition across biological sources. However, cross-recognition, cross-sensitization, and clinically relevant cross-allergy are distinct. Structural homology creates potential for cross-recognition, whereas clinical outcomes also depend on IgE affinity, dose, epitope density, gastrointestinal transformation and absorption, and individual response thresholds. In clinical practice, precision allergy molecular diagnostic applications (PAMD@) can help distinguish clinically relevant sensitization from cross-reactivity, thereby supporting individual risk assessment [45]. Structural and processing studies should specify whether they assess IgE binding, effector-cell activity, or clinical elicitation [4,5]. ‘Antigenicity’ is used only when a source study refers to broader antibody recognition.

### 2.4. Protein Stability, Disulfide Networks, and Aggregation

Protein stability links native architecture to processing-induced transformations, yet is a multidimensional property rather than a single uniform trait. Thermal stability, chemical stability, proteolytic resistance, retention of tertiary structure, solubility, and preservation of IgE-reactive regions do not necessarily change in parallel. A protein may lose its native fold while retaining immunoreactive fragments, or remain largely intact while surface modifications alter recognition. Accordingly, studies should specify the stability dimension, testing conditions, and assessed endpoint [2,3,25].

Disulfide bonds are major covalent constraints on allergen stability. In 2S albumins and nsLTPs, disulfide networks restrict flexibility and can preserve structural domains under stress, but bond number alone is insufficient. The effect of a disulfide network also depends on its connectivity and structural context, including solvent accessibility, proximity to cleavage sites, and its relationship to epitopes. Heating can further drive disulfide exchange and generate states distinct from both native and fully reduced proteins [2,3,27,29,30].

Oligomerization adds another level of structural complexity, with cupins often forming multimers whose interfaces shield some regions and stabilize others. Heating can dissociate native oligomers and expose hydrophobic surfaces that promote non-native association, making quaternary structure part of the processing response rather than a fixed property.

Aggregation can decrease, preserve, or increase immunoreactivity while altering analytical recovery. Heat-induced unfolding exposes hydrophobic and chemically reactive regions that promote intermolecular association, disulfide exchange, and covalent cross-linking. Depending on the structure of the aggregate, antibody-binding regions may become masked and solubility reduced, lowering the apparent concentrations measured by ELISA; aggregation may also preserve digestion-resistant regions, expose previously buried sequences, or increase local IgE-binding-site density and multivalency [6,7]. The physical properties of aggregates and their intermolecular bonding further influence biological accessibility and analytical recovery. Large insoluble aggregates may be poorly extractable, whereas smaller soluble aggregates remain accessible to antibodies and digestive enzymes. Reduced levels of soluble IgE-reactive protein may therefore reflect loss during sedimentation or extraction rather than irreversible epitope loss.

### 2.5. Gastrointestinal Stability as Structural Context

Processing and matrix interactions shape an allergen’s molecular state before digestion begins. Gastrointestinal conditions then act on that pre-existing state, so digestive behavior cannot be inferred from the native protein alone.

Protein architecture influences protease accessibility and the persistence of allergen-derived species [2,3,42,46]. Digestion may leave structured fragments or molecular assemblies; the identity and molecular state of the remaining species are more informative than survival of the native protein alone. Table 1 summarizes the major allergen families and their structural features.

## 3. Processing-Induced Molecular Remodeling of Food Allergens

Food processing modifies food allergens through interconnected physical and chemical events. Since the resulting molecular changes depend on allergen identity, matrix composition, processing conditions, and molecular state, measured immunoreactivity may decrease, be preserved, or increase [6,7,8,23,48].

### 3.1. Thermal Processing: Denaturation, Aggregation, and Epitope Redistribution

Thermal processing can disrupt the noncovalent interactions that stabilize protein structure, and the resulting molecular state is determined by heating conditions and the surrounding matrix. Partially unfolded proteins may either refold or undergo irreversible denaturation, fragmentation, or aggregation [6,7,21]. Thermal effects depend on allergen architecture. Loss of native folding may reduce conformational epitope recognition, whereas disulfide-rich allergens such as 2S albumins and nsLTPs can retain organized regions and IgE reactivity after heating. Intrinsically disordered proteins such as caseins may similarly retain linear IgE-reactive regions even after extensive heating.

Thermally induced unfolding can promote aggregation through noncovalent association, disulfide exchange, and covalent cross-linking. The resulting aggregates may either mask or expose IgE-reactive regions, and alter protein solubility and extractability. Therefore, reduced soluble protein or immunoassay recovery after heating does not necessarily indicate a proportional reduction in biologically relevant allergenic material.

The heating environment further shapes these outcomes. Boiling may transfer soluble allergens into cooking water, whereas baking and roasting combine high temperature with decreasing water activity and favor aggregation, cross-linking, and chemical modification. For peanut 2S albumins, aqueous heating and heating–glycation can reduce IgE reactivity under defined experimental conditions, whereas Ara h 2 and Ara h 6 isolated from roasted peanut retained native-like IgE reactivity in one study [49]. These contrasting observations indicate that processing temperature alone cannot predict allergenic outcomes; the surrounding matrix and the molecular state present during heating are also important determining factors.

### 3.2. High-Pressure Processing: Reversible and Irreversible Conformational Changes

High-pressure processing (HPP) inactivates microorganisms while limiting some changes associated with intensive heating. Pressure primarily perturbs noncovalent interactions, hydration, and molecular packing, while leaving peptide bonds and most covalent structures intact. These perturbations can reorganize tertiary and quaternary protein structures, producing either reversible conformational changes or persistent aggregation [6,50]. Therefore, HPP outcomes depend on the pressure–time–temperature history and the surrounding physicochemical and matrix environment. Moderate pressure may generate intermediates that refold after decompression, whereas stronger treatment can cause persistent rearrangement or aggregation.

In β-lactoglobulin, pressure-induced conformational changes are accompanied by a non-monotonic IgE-binding response: binding decreases up to 200 MPa, rises between 200 and 400 MPa, and declines again as pressure approaches 600 MPa [51]. However, at 60 °C, IgE binding declines continuously with increasing pressure. This contrasting response provides a concrete example of why pressure-induced structural changes do not translate into uniform changes in IgE recognition.

Accordingly, HPP-induced changes in IgE recognition should be interpreted relative to the matrix context, protein stabilization, and the epitope repertoire represented in the assay, rather than pressure intensity alone [6,50].

### 3.3. Fermentation and Enzymatic Hydrolysis: Proteolysis and Fragment Persistence

Fermentation can modify allergenic proteins through microbial proteolysis, acidification, redox changes, and interactions with fermentation-derived metabolites. The extent and location of cleavage depend on the microbial strain, substrate composition, fermentation conditions, and protease specificity. The disappearance of the intact parent protein or its electrophoretic band does not by itself confirm loss of IgE recognition [6,40]. Limited proteolysis can leave IgE-binding epitopes or assemblies capable of cross-linking FcεRI-bound IgE, while compact folding or disulfide connectivity can restrict protease access to cleavage sites. The resulting fragment profile therefore reflects the interplay among protease specificity, the extent of hydrolysis, and the structural context of cleavage sites.

Experimental observations with β-lactoglobulin illustrate the distinction between degradation and loss of IgE recognition. During lactobacillus-mediated milk fermentation, β-lactoglobulin was progressively degraded while overall IgE binding changed comparatively little under the tested conditions. More extensive tryptic hydrolysis, in contrast, substantially reduced both intact protein and IgE-binding capacity [40]. Protein degradation and loss of IgE recognition are related but non-equivalent endpoints. Controlled enzymatic hydrolysis can be tuned through enzyme selection and reaction conditions to adjust cleavage patterns and the extent of hydrolysis. Extensive hydrolysis may reduce the abundance and multivalency of IgE-reactive species but compromise ingredient functionality, whereas incomplete hydrolysis may leave reactive sequences or persistent fragments intact. Hydrolysates should therefore be assessed using complementary molecular and functional endpoints, including IgE binding and, where feasible, effector-cell activity.

### 3.4. Glycation and Maillard Chemistry: Epitope Masking and Modified Molecular Surfaces

Maillard chemistry begins when protein amino groups react with reducing sugars. Schiff-base formation and Amadori rearrangement are followed by dehydration, oxidation, fragmentation, and cross-linking that generate heterogeneous advanced glycation products. These reactions can modify susceptible residues, alter charge and hydrophobicity, and form intra- or intermolecular cross-links. Outcomes depend strongly on reaction conditions, sugar chemistry, protein conformation, and matrix composition [6,7,49,52].

Glycation may reduce IgE binding by chemically modifying residues within an epitope, sterically restricting antibody access, destabilizing conformational epitopes, or incorporating reactive regions into larger aggregates. For Ara h 2 and Ara h 6, heating–glycation reduced IgE-binding capacity relative to heating alone under the tested solution-processing conditions [49]. This finding illustrates an experimentally defined molecular outcome rather than a universal effect of Maillard chemistry across food allergens.

Opposite outcomes are also possible. Partial unfolding during glycation may expose previously buried sequences, whereas cross-linking can increase molecular size and enhance the multivalent presentation of IgE-binding regions. Newly generated chemical structures may additionally alter antigen uptake or interactions with receptors that recognize advanced glycation products. Studies of roasted peanuts show that processing within the food matrix and multiple protein modifications complicate attribution of in vitro immunological readouts to glycation alone [53,54]. The attribution of altered IgE recognition to a single chemical pathway therefore requires direct molecular evidence.

### 3.5. Oxidation and Other Chemical Modifications

Oxidative modification can arise during thermal and non-thermal processing and storage. Reactive oxygen and nitrogen species can modify susceptible residues, generate carbonyl groups, and alter disulfide connectivity, leading to downstream effects on protein cross-linking, surface properties, aggregation, and protease accessibility.

Proteomic analysis of peanut allergens has shown that roasting alters the profiles of post-translational modifications. For example, methionine oxidation occurs frequently, and multiple types of modifications are only detected in roasted samples. Nevertheless, raw and roasted extracts did not differ significantly in pooled-serum IgE binding by enzyme-linked immunosorbent assay (ELISA) in that study [54]. Extensive chemical remodeling can therefore occur without producing a measurable change in a particular immunological assay.

Cold plasma provides a contrasting example, because reactive species can be generated at relatively low bulk temperatures. Under the conditions, plasma treatment of shrimp tropomyosin altered secondary structure and surface hydrophobicity and reduced both IgE binding and cellular degranulation-related responses [55]. This finding illustrates an allergen- and condition-specific response rather than a general effect of plasma treatment across food allergens. Because the effects of plasma treatment depend on treatment chemistry and intensity, matrix context, and the molecular target, their evaluation should consider residual IgE recognition and functional activity alongside overall product quality.

### 3.6. IgE Recognition and Biological Activity After Processing

Processing can alter IgE recognition through opposing effects on epitope integrity, accessibility, and multivalency, while fragmentation may further uncouple IgE binding from FcεRI cross-linking. Therefore, the same process can yield different outcomes across allergens and food systems.

Interpretation depends on the endpoint measured. Assays using analytical antibodies report on the recognition of defined targets, whereas tests using patient sera assess IgE binding under the tested conditions. Basophil and mast-cell assays assess effector-cell activation, and clinical challenge studies evaluate allergic elicitation in sensitized individuals. These endpoints are related but not interchangeable, and terminology should reflect the evidence supporting each conclusion [4,5].

Wheat illustrates the distinction between an analytical target and a clinically relevant allergen. Processing alters gluten-protein assembly and analytical recovery [22,56], but generic gluten detection alone cannot assess the clinical relevance of ω-5 gliadin in wheat-dependent exercise-induced anaphylaxis [41].

**Figure 2:**
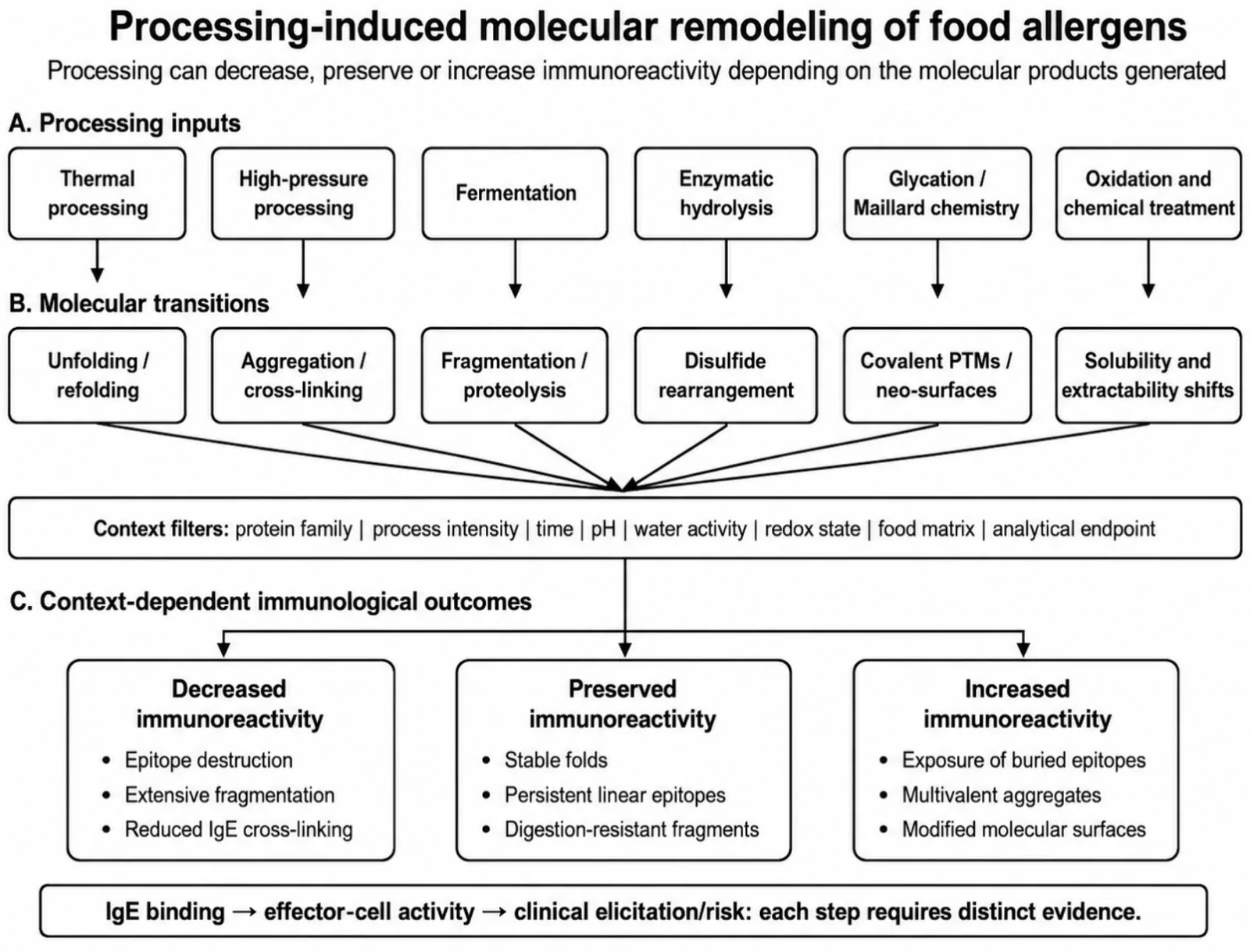
Processing-induced molecular remodeling and context-dependent changes in allergen immunoreactivity. Thermal processing, high-pressure processing, fermentation, enzymatic hydrolysis, glycation and Maillard chemistry, and oxidation can induce unfolding, refolding, aggregation, fragmentation, disulfide rearrangement, covalent modification, and changes in solubility or extractability. The balance among these events may decrease, preserve, or increase IgE recognition and biological activity. The direction and magnitude of change depend on allergen architecture, process intensity, pH, water activity, redox conditions, food matrix composition, and the endpoint measured. Abbreviations: IgE, immunoglobulin E; PTMs, post-translational modifications.

Therefore, processing is better understood as context-dependent molecular remodeling rather than an intervention with a predictable allergenic outcome [8,9,42]. Figure 2 and Table 2 summarize the principal determinants and endpoints.

## 4. Food Matrix Effects on Allergen Structure, Accessibility, and Immunoreactivity

Processing outcomes depend not only on allergen architecture but also on the molecular and physical environment in which structural and chemical transformations occur. Food matrix components, including lipids, carbohydrates, polyphenols, minerals, salts, and other proteins, shape the local physicochemical conditions and food microstructure. By altering ionic conditions, water availability, molecular crowding, and interfacial organization, these components can modify protein folding, aggregation, chemical reactivity, digestion, and analytical recovery [11,12,13]. Matrix effects are bidirectional and context-dependent, varying with allergen identity, component concentration, processing history, and food microstructure. Results obtained with purified proteins should therefore not be extrapolated directly to intact processed foods without taking both matrix composition and physical organization into account [11,12,13,42,57,58].

### 4.1. Food Microstructure and Physical Organization

Food microstructure governs the access of allergenic proteins to processing reagents, digestive enzymes, antibodies, and extraction solvents. Dense structures can restrict diffusion and delay release, whereas mechanical disruption and emulsification can increase the exposed surface area and intermolecular contact. Reduced water mobility may favor aggregation and cross-linking, while oil–water interfaces can alter conformation and epitope presentation. Matrix effects therefore depend on both bulk composition and physical organization [11,12,13].

### 4.2. Lipid–Allergen Interactions and Interfacial Organization

Nonspecific lipid transfer proteins (nsLTPs) provide a clear example of direct ligand-dependent effects. Their hydrophobic cavities can bind fatty acids, which may alter molecular flexibility and the accessibility of surface-exposed regions. In Pru p 3, oleic acid binding induced conformational changes near a major IgE epitope and increased both serum IgE binding and basophil activation, whereas stearic acid did not materially affect either endpoint under the tested conditions [33]. Lipid ligands also modify the stability and IgE recognition of Len c 3 [32], illustrating that apo-allergens and ligand-bound forms are not necessarily immunologically equivalent.

Lipids can also influence allergens through food microstructure rather than direct ligand binding. Association with lipid droplets, membranes, or emulsified interfaces can alter protein release, conformation, protease access, and analytical recovery. During heating, interfacial layers may reorganize into mixed assemblies containing allergens, other proteins, phospholipids, and surfactants, making lipid composition and supramolecular organization additional determinants of allergen behavior [3,11].

### 4.3. Carbohydrates and Water Activity

Carbohydrates can modify the physicochemical environment of allergenic proteins by altering water activity, molecular mobility, phase organization, and chemical reactivity during processing. Reducing sugars participate in Maillard chemistry, whereas polysaccharide and hydrocolloid networks can restrict protein mobility and the diffusion of enzymes or extraction solvents [11,12,13]. In baked foods, cellular structures and thermally generated carbohydrate–protein networks may physically entrap allergenic proteins without chemically eliminating them.

Water activity is particularly important because it alters both molecular mobility and chemical reaction pathways. Low water availability can favor aggregation, cross-linking, and Maillard reactions under appropriate processing conditions, whereas greater water availability facilitates molecular diffusion, hydrolysis, and protease access. The same allergen may therefore undergo different combinations of unfolding, aggregation, glycation, and fragmentation in solution, dough, emulsions, or low-moisture powders [11,12,13].

### 4.4. Polyphenols

Polyphenols interact with proteins through hydrogen bonding, hydrophobic contacts, and π–π interactions and, after oxidation to quinones, through covalent reactions with nucleophilic amino acid side chains. These interactions may alter protein conformation, hide antibody-accessible regions, encourage precipitation or cross-linking, and modify protease accessibility [12].

Analytical and biological consequences of polyphenol interactions should be distinguished. Precipitation or cross-linking can reduce extraction and immunoassay recovery even when immunologically relevant species remain, whereas conformational changes may genuinely alter IgE recognition or digestion. Therefore, lower immunochemical recovery does not by itself establish reduced biological activity or clinical elicitation [12].

### 4.5. Mineral Ions and Small-Molecule Ligands

Mineral ions can influence allergen conformation through site-specific coordination, electrostatic screening, and stabilization of ligand-dependent structural states. Calcium occupancy provides a representative example. Depletion of protein-bound Ca^2+^ substantially reduced IgE binding to cross-reactive fish parvalbumins, consistent with calcium-sensitive epitope organization [35]. α-Lactalbumin likewise exists in calcium-bound and calcium-depleted conformational states; experimentally altering calcium occupancy has been associated with changes in protein structure, antibody recognition, and basophil degranulation-related readouts [59].

These examples show that ligand occupancy can alter molecular state and immune recognition, but the direction and magnitude depend on the specific protein, ligand, structural state, and endpoint.

### 4.6. Protein–Protein Interactions and Supramolecular Networks

Proteins in complex foods do not behave independently. Ionic strength, pH, protein concentration, and processing history govern solubility and intermolecular association, while processing can promote mixed aggregates and supramolecular networks.

Milk provides a representative example. Heating promotes association of unfolded β-lactoglobulin with κ-casein through disulfide exchange and noncovalent interactions, producing mixed complexes and larger aggregates [18,47]. Such assemblies can alter protein solubility, extractability, protease accessibility, and the exposure of antibody-binding regions.

More broadly, protein–protein association can change aggregate size and access by digestive enzymes or analytical reagents. Supramolecular organization is therefore part of the matrix-conditioned allergen state.

### 4.7. Integrated Effects on Processing, Gastrointestinal Fate, and Analytical Recovery

Matrix components act simultaneously during processing and gastrointestinal transformation. Lipids, sugars, polyphenols, mineral ions, and proteins jointly influence hydration, heat transfer, redox chemistry, molecular mobility, aggregation, and allergen release. Peanut-matrix studies show disulfide-rich 2S albumins substantially contribute to IgE reactivity of persistent digestion products [28]. Across peanut, baked egg/peanut, dairy, and egg systems, matrix context and digestion conditions modify allergen bioaccessibility and peptide profiles, rather than producing a uniform protective or delaying effect [11,28,53,60].

The same matrix properties also affect analytical measurements by altering extraction, target accessibility, and downstream recovery. Immunoassays can therefore underestimate molecular species with masked, modified, or poorly extractable target epitopes [9,10,58]. Mass spectrometry is less dependent on intact, antibody-accessible epitopes but remains sensitive to matrix effects on extraction, digestion, peptide recovery, and ionization [9,10,43,48,61]. Variations between analytical platforms should be interpreted based on their measurands and sample preparation workflows, rather than treated simply as method inconsistency.

Matrix-related analytical bias can be reduced through matrix-appropriate calibration, recovery assessment, and orthogonal validation, although no single strategy can eliminate all sources of uncertainty. Method performance should therefore be validated for the relevant allergenic source, processing history, molecular target, and food matrix. Overall, the food matrix connects allergen architecture, processing-induced remodeling, gastrointestinal persistence, and analytical recovery within a coupled molecular system. Future studies should characterize both matrix composition and physical structure, compare purified-protein and whole-food systems under matched processing conditions, and, where feasible, integrate structural, proteomic, IgE-binding, and functional endpoints using the same processed materials [11,12,13]. The principal categories of matrix–allergen interaction and their implications for gastrointestinal fate and analytical recovery are summarized in Table 3.

## 5. Gastrointestinal Transformation and Allergen Persistence

After being released from the food matrix, allergen-derived molecular species undergo sequential oral, gastric, and intestinal transformations under changing physicochemical and proteolytic conditions. These processes generate heterogeneous mixtures of intact proteins, aggregates, disulfide-linked fragments, and shorter peptides. Gastrointestinal digestion is therefore better viewed as a molecular selection and transformation process than as a binary transition between intact and fully degraded proteins. Its immunological relevance depends on the abundance and molecular organization of the persisting species, including their solubility, epitope content, connectivity, and multivalency [3,46,60,62,63,64].

### 5.1. Oral Release and Gastric Transformation

Gastrointestinal transformation begins with release of allergenic proteins from the food matrix. Mastication increases exposed surface area and mixing with saliva, while bioaccessibility depends on particle size, porosity, solubility, and protein–matrix interactions. Here, bioaccessibility denotes accessibility within the gastrointestinal environment and is distinct from epithelial transport or systemic bioavailability. In the stomach, acidification can alter conformation, expose pepsin cleavage sites, or promote aggregation; apparent digestion resistance therefore reflects the gastric environment and matrix context rather than protein sequence alone [3,63,64].

Host physiology also shapes gastric transformation. Gastric pH, enzyme activity, and other gastrointestinal conditions differ among groups such as infants and adults, altering digestion products. Food buffering, lipid content, and structure can further affect acidification, gastric emptying, and enzyme diffusion [63,64,65].

### 5.2. Duodenal and Intestinal Transformation

On entering the duodenum, gastric digesta are neutralized and exposed to bile salts, phospholipids, and pancreatic enzymes. The shift toward neutral pH can induce refolding, precipitation, dissociation, or further aggregation, altering cleavage-site accessibility and the molecular species that persist. Pancreatic proteolysis depends on both enzyme specificity and structural accessibility; compact folding, aggregation, ligand binding, glycosylation, or disulfide bonding can protect sites that are otherwise susceptible. Bile salts can further alter conformation, aggregation, and protease access through interactions with hydrophobic surfaces, with protein- and condition-dependent effects.

Pru p 3 provides an illustrative example. In the presence of bile salts, intestinal proteolysis was substantially accelerated and generated approximately 100 peptides, many of which remained connected through disulfide bonds to form persistent structures of approximately 5–6 kDa [66]. In the bile-salt digest, immunoblotting showed no detectable IgE reactivity, whereas inhibition ELISA detected residual IgE binding in the tested sera [66]. Extensive peptide-bond cleavage therefore does not necessarily yield fully independent or immunologically inactive fragments. Digestion is sequential: gastric cleavage may expose pancreatic protease sites, whereas gastric aggregation can restrict later access. Persistence studies are therefore most informative when they preserve the physiological sequence of gastric and intestinal digestion.

### 5.3. Digestion-Resistant Fragments and Residual IgE Reactivity

Persistent digestion products range from intact proteins and structured assemblies to shorter IgE- or T-cell-reactive peptides, and their biological significance depends on the specific molecular state and examined endpoint.

However, IgE binding alone does not establish functional activity. Short or effectively monovalent peptides may retain IgE recognition but have limited capacity to cross-link FcεRI-bound IgE efficiently. In contrast, larger fragments, disulfide-linked assemblies, or aggregates may retain greater molecular multivalency and therefore have a higher potential to activate basophils or mast cells [2,3]. This relationship depends on epitope number, spacing, and accessibility, as well as the IgE repertoire examined.

Limited cleavage can also alter functional readouts in ways that are not predicted by loss of intact protein alone. After pepsin digestion in vitro, Ara h 1 and Ara h 3 showed increased mast-cell activation under some experimental conditions, whereas Ara h 2 and Ara h 6 were less affected [67]. These observations indicate that digestion can generate molecular states with altered functional activity rather than simply eliminating immunological recognition progressively. Peptides that no longer support efficient IgE cross-linking may still retain T-cell-reactive sequences and participate in subsequent antigen processing. Persistent digestion products should therefore be characterized according to the biological endpoint under investigation rather than classified solely by peptide size or disappearance of the parent protein.

### 5.4. Epithelial Accessibility and Transport

To interact with immune compartments beyond the lumen, allergen-derived species must reach the mucus layer and epithelial surface and may then undergo uptake or transport. Epithelial accessibility and transport are distinct because material can reach the surface without crossing the barrier.

Epithelial accessibility and transport depend on the molecular and supramolecular properties, matrix context, epithelial-barrier characteristics, and intracellular processing. Transport is therefore not simple size-based filtration, and detection of transported material does not establish systemic bioavailability, effector-cell activation, or clinical elicitation.

Experimental comparisons among peanut allergens illustrate the importance of allergen-specific molecular behavior. In an epithelial-transport model, Ara h 1 and Ara h 3 showed reduced functional activity after transport, whereas Ara h 2 and Ara h 6 retained comparatively greater activity [67]. These differences are consistent with the structurally constrained, disulfide-rich architecture of 2S albumins while also demonstrating that digestive persistence and persistence of functional activity after epithelial transport are related but non-equivalent properties.

Matrix-mediated changes in allergen release, digestion, and epithelial accessibility should likewise not be interpreted uniformly as protective [8,11,12]. Delayed release may change the temporal profile of luminal exposure, whereas fragmentation may increase the abundance of smaller, potentially transportable molecular species while reducing the multivalency required for efficient IgE cross-linking. The biological significance of either outcome depends on the molecular species generated and the endpoint measured.

Epithelial culture systems provide useful mechanistic information, but transport data require additional functional characterization to determine whether the transported species retain IgE-binding or effector-cell activity. Standardized molecular and functional endpoints would improve the interpretation of these findings.

### 5.5. Experimental Models and Interpretation of Digestive Stability

Early digestion assays commonly exposed purified proteins to fixed simulated gastric conditions and monitored disappearance of intact protein bands using sodium dodecyl sulfate–polyacrylamide gel electrophoresis (SDS–PAGE) [62,63,64]. Such results are highly sensitive to digestion conditions, protein loading, sampling strategies, and analytical detection limits [63,64].

The INFOGEST framework improves experimental comparability by providing standardized protocols for the oral, gastric, and intestinal phases under defined digestion conditions [63,64,65]. Nevertheless, a static adult digestion model cannot reproduce all physiological processes, including dynamic gastric emptying, continuous secretion, nutrient absorption, microbiota activity, or population-specific gastrointestinal physiology. Results obtained in static digestion systems should therefore be interpreted as standardized experimental approximations rather than direct simulations of clinical exposure.

Analytical resolution also determines which digestion products are observed. Gel electrophoresis primarily captures intact proteins and relatively large fragments, whereas LC-HRMS can resolve smaller peptides and sequence-specific modifications. Immunoassays and effector-cell assays provide complementary information regarding antibody recognition and residual functional activity [9,10,43,68].

Robust digestion studies should combine characterization of molecular persistence and fragment identity with IgE-binding and, where feasible, functional endpoints under relevant processing, matrix, and simulated host conditions [3,9,10,62,63,68].

**Figure 3:**
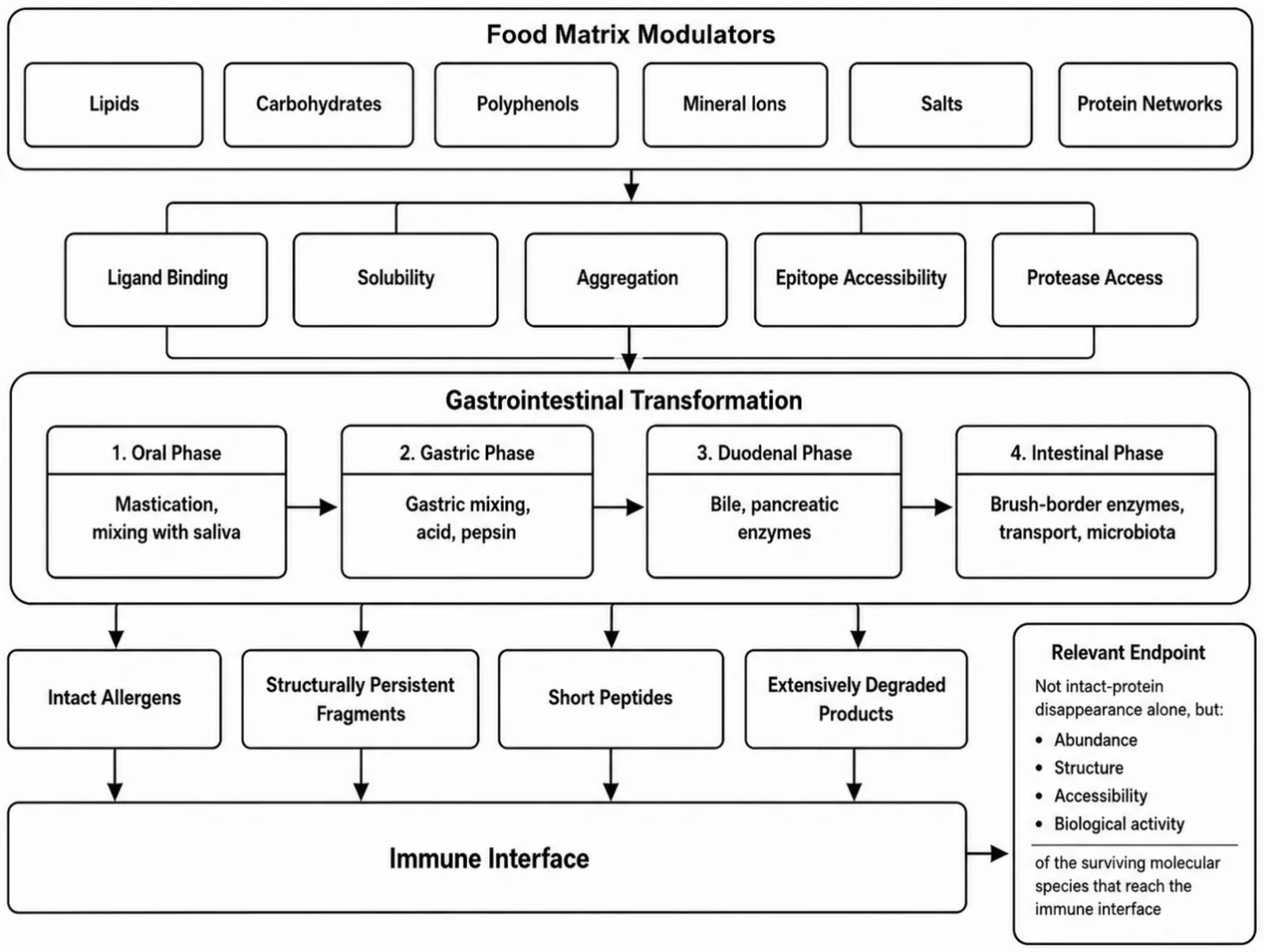
Food matrix modulation and gastrointestinal transformation of food allergens. Lipids, carbohydrates, polyphenols, mineral ions, salts, and protein networks can alter ligand binding, solubility, aggregation, epitope accessibility, and protease access. During oral, gastric, duodenal, and intestinal phases, these matrix-conditioned properties influence whether intact allergens, structurally persistent fragments, disulfide-linked assemblies, short peptides, or extensively degraded products remain accessible at the intestinal immune interface. The relevant endpoint is not disappearance of the intact parent protein alone, but the abundance, molecular structure, accessibility, and biological activity of the species that persist.

Digestive stability is an exposure-related property rather than an independent predictor of clinical elicitation. Persistent intermediates may remain relevant when they retain structural integrity, accessible epitopes, and multivalency, but persistence alone does not establish biological significance and requires endpoint-matched evidence. Figure 3 summarizes matrix-conditioned gastrointestinal transformation and the species that may reach the intestinal immune interface.

## 6. Detection Technologies for Structurally Dynamic Food Allergens

Reliable food-allergen detection depends on more than analytical sensitivity. The reported results reflect the entire analytical workflow, from sampling and extraction to target recognition, quantification, and interpretation, all of which can be affected by processing and matrix effects. Allergenic-source proteins may remain present yet become poorly soluble, modified, fragmented, aggregated, or inaccessible to the method. A reduced signal therefore does not necessarily indicate absence of allergenic-source protein or lower biological activity.

Analytical platforms interrogate different measurands. Immunoassays measure accessible antibody-binding epitopes, PCR-based methods detect source-specific DNA, mass spectrometry measures selected allergen-derived peptides or broader proteomic signatures, and biosensors report recognition-dependent signals. These outputs are not interchangeable measures of intact allergen abundance, IgE-binding capacity, effector-cell activity, or clinical risk. Method selection should therefore be guided by the intended analytical use, whether for screening, source identification, quantification, molecular characterization, or exposure assessment.

### 6.1. Target Selection, Sampling, and Extraction

The analytical workflow begins before molecular detection. Cross-contact may be heterogeneous within a batch, especially for particulate materials such as nuts, seeds, powders, or baked fragments. Sampling design, test-portion size, homogenization, subsampling, and replicate analysis are therefore integral to reliability.

Extraction is another major source of analytical variability. The protein recovery depends on extraction chemistry, extraction conditions, and sample-to-buffer relationships, all of which interact with matrix composition and processing state. Lipid-rich foods may require defatting or detergent-assisted extraction, whereas polyphenol-rich matrices may require conditions that limit precipitation or nonspecific protein interactions. Heat-processed foods can contain poorly soluble aggregates and covalently cross-linked protein networks that are incompletely recovered by standard aqueous buffers. Increasing extraction stringency may improve protein recovery but can also alter the molecular structures or epitopes required for immunochemical recognition.

Target selection should reflect processing state. Native protein targets may suit minimally processed foods, whereas validated linear epitopes or proteotypic peptides may be more robust after denaturation, aggregation, fermentation, hydrolysis, or chemical modification. Sequence conservation within allergen families can also promote antibody cross-reactivity or yield peptide markers with insufficient source specificity.

The analytical measurand should be explicit. Results may represent a specific allergen protein, total allergenic-source protein, ingredient mass, DNA copy number, peptide concentration, or an assay-specific response. Conversion among these quantities requires validated relationships; “ppm allergen” is ambiguous unless the reported quantity is defined.

### 6.2. Immunochemical Methods

ELISAs remain widely used for food-allergen screening and quantification because they are sensitive, relatively inexpensive, and high throughput. Sandwich formats use capture and detection antibodies against accessible target regions, whereas competitive formats can accommodate fragmented or structurally altered targets that cannot support simultaneous recognition by two antibodies.

Immunoassay performance depends on both antibody specificity and accessibility of the recognized epitope. Antibodies directed against conformational epitopes may lose reactivity when processing disrupts the native protein fold, whereas antibodies recognizing linear epitopes may retain recognition of denatured proteins or persistent fragments. Aggregation can mask target epitopes, restrict antibody access, or alter apparent binding through changes in molecular presentation. Glycation, oxidation, and proteolysis can also modify residues within the assay epitope while leaving other IgE-reactive molecular regions intact [9,10,58,68].

Commercial immunoassays for the same source may differ because they recognize different proteins, epitopes, or processed forms. ELISA and LC–MS/MS can also diverge because they interrogate different measurands and respond differently to extraction, digestion, and matrix interference. Such discordance should be interpreted as target-, method-, and matrix-dependent rather than evidence that one platform is universally superior [9,10,61].

Representative incurred materials are preferred for method evaluation because the allergenic ingredient undergoes processing with the food matrix. Spiked finished products do not reproduce these transformations and may overestimate recovery or underestimate processing- and matrix-associated bias.

### 6.3. DNA-Based Detection

PCR-based methods, including conventional PCR, qPCR, and dPCR, identify allergenic food sources through source-specific DNA sequences [69,70]. DNA-based methods can achieve high analytical specificity and are particularly useful for source confirmation when protein structures have been extensively modified or when suitable antibody-based assays are unavailable. Digital PCR has been applied to sesame detection, while a multiplex digital PCR assay evaluated multiple DNA targets for peanut detection [71,72].

Digital PCR enables the absolute or near-absolute quantification of source-specific DNA through reaction partitioning and can improve measurement at low target abundance without reliance on external calibration curves. Droplet digital PCR has been applied to peanut-source detection in food ingredients, although DNA copy number cannot be directly converted into the concentration of peanut protein [72]. Nanoplate digital PCR has likewise been applied to trace detection of sesame DNA in processed foods [71].

DNA is not the IgE-active molecular species. Relationships among DNA, ingredient mass, total source protein, and individual allergens vary with species, tissue, processing, and formulation. DNA may persist after protein degradation, while refined ingredients may retain protein with little recoverable DNA. PCR therefore supports source identification rather than direct exposure measurement.

### 6.4. Mass Spectrometry-Based Proteomics

Mass spectrometry enables sequence-specific detection of allergen-derived peptides and can support simultaneous analysis of multiple allergenic sources. In bottom-up workflows, extracted proteins are enzymatically digested, and selected proteotypic peptides are used as surrogate markers for target proteins or allergenic sources [9,10,61,68].

Marker-peptide selection is critical. Suitable marker peptides should combine source or protein specificity with reproducible generation and analytical stability across relevant biological variability. Peptides containing residues that are particularly susceptible to processing-induced modification may show variable recovery or signal intensity. Conversely, modified peptides can be targeted deliberately when the objective is to characterize oxidation, glycation, deamidation, or other processing-derived molecular changes.

Mass spectrometry is less dependent than immunoassays on preservation of intact protein conformation but is not independent of processing effects. Aggregation and covalent cross-linking can reduce the efficiency of both extraction and enzymatic digestion. Maillard modification of lysine or arginine may interfere with tryptic cleavage. Oxidation, other chemical modifications, or sequence variation can alter peptide mass, chromatographic behavior, or fragment-ion intensity. Extraction and digestion recovery require validation in the relevant processed matrices [9,10,61].

Matrix interference remains a key analytical challenge. Co-extracted peptides, lipids, carbohydrates, salts, and other food components can suppress ionization or generate interfering signals and overlapping transitions. Chromatographic separation, scheduled acquisition, qualifier transitions, appropriate blank matrices, and interference-monitoring strategies can improve selectivity, but targeted LC–MS/MS methods still require matrix-specific evaluation rather than assuming equivalent performance across products [9,10,61,68].

Quantitative analysis also requires appropriate calibration and internal standards. Stable-isotope-labeled peptides can correct effectively for variability introduced during chromatography, ionization, and instrumental detection, but they do not fully account for protein losses during extraction or enzymatic digestion when introduced only after these steps. Isotope-labeled proteins or other internal standards added earlier in the workflow can provide broader process control, although they are more difficult and costly to produce and characterize.

A further challenge is conversion from marker-peptide concentration to total protein from the allergenic source. The abundance of a selected marker protein can vary within the source ingredient, and recovery of a single peptide does not necessarily represent all allergenic proteins from that source. Quantitative methods intended to support exposure assessment should use experimentally validated marker-to-total-protein conversion factors and state explicitly whether results refer to a specific target protein or to total protein from the allergenic source. Targeted LC–MS/MS methods have been validated for quantifying peanut-, egg-, and milk-derived protein in fortified and incurred food matrices [73].

### 6.5. Biosensors, Nanomaterials, and Microfluidic Platforms

Biosensor platforms couple molecular-recognition elements with physicochemical transducers to convert allergen recognition into measurable signals. Antibodies, aptamers, nucleic acid probes, and synthetic receptors have been combined with electrochemical or optical sensing formats, while nanostructured materials can enhance surface area or boost signal amplification [61,68,74,75]. Nanozyme-based systems may provide catalytic signal amplification, although their analytical robustness and practical scalability still remain to be established [76].

Biosensors and microfluidic platforms may support rapid screening and process-line testing because of short analysis times and portability. Ambiguous results can be evaluated by validated laboratory methods. Wider routine or regulatory adoption requires reproducible manufacture, reagent stability, quality control, interlaboratory validation, matrix-specific performance, and clear decision criteria.

### 6.6. Method Validation and Molecular Interpretation

Analytical sensitivity alone is insufficient to determine whether a method is suitable. Validation should address specificity, selectivity, recovery, repeatability, intermediate precision, robustness, and linearity. Limits of detection and quantification, measurement uncertainty, and matrix interference should also be evaluated. When performance is strongly matrix- or process-dependent, these parameters should be evaluated across representative food categories and relevant processing conditions.

Orthogonal methods are useful when processing remodels the target protein. Agreement between methods with distinct measurands provides complementary evidence, whereas divergence can reveal mechanism. For example, ELISA-negative/LC–MS/MS-positive results may reflect epitope masking, extraction differences, or preserved marker peptides; PCR-positive/protein-negative results may reflect persistent DNA, poor protein recovery, degradation, or sensitivity differences. Such discrepancies warrant mechanistic investigation.

For exposure assessment, results should preferably be reported as total allergenic-source protein per unit mass of food. Conversion from the primary measurand requires validation, and reports should specify target, extraction, recovery, calibration, conversion factor, uncertainty, and matrix.

Analytical detection does not directly determine whether a given exposure will elicit clinical symptoms, although reliable quantification remains essential for estimating allergenic-source protein exposure and comparing it with reference doses.

Source-specific validation is particularly important when an allergenic food contains heterogeneous molecular targets. In wheat, α/γ-gliadins have been investigated as immunochemical targets, whereas ω-5 gliadin is clinically relevant in wheat-dependent exercise-induced anaphylaxis. These are distinct analytical and clinical questions [41,56]. Sesame methods must account for multiple allergenic proteins and processing-dependent recovery [20,42,71], while soybean assays likewise require explicit target selection, as illustrated by glycinin-specific immunochemical detection [21,77].

Method validation should therefore be tailored to the specific measurand and the intended analytical purpose. Table 4 summarizes the principal limitations and appropriate applications of each analytical approach.

## 7. Molecular Risk Assessment and Interpretation

Risk assessment becomes relevant when molecular measurements are interpreted within a dose–exposure framework. Molecular detection establishes the potential for exposure but does not quantify risk, which depends on the consumed dose of allergenic-source protein, population eliciting-dose distributions, and uncertainties in contamination, sampling, and analytical conversion. Molecular risk interpretation links these variables to exposure estimates, reference-dose comparison, and risk-management decisions [80]. In composite foods, source attribution may be complicated when an allergen derives either from an intentionally added ingredient or from unintended cross-contact [81].

### 7.1. From Molecular Detection to Exposure Assessment

Quantitative exposure assessment requires a defined measurand and reporting unit. For comparison with clinical threshold distributions or reference doses, results are preferably expressed as total protein from the allergenic source per unit mass of food. Marker-based or assay-specific measurands can be converted to total allergenic-source protein through validated relationships that account for target abundance, analytical recovery, calibration, and processing effects.

For homogeneous foods, the ingested dose of allergenic-source protein can be approximated as

*D* = *C* × *P*

where *D* is the consumed dose of allergenic-source protein (mg), *C* is its concentration in the food (mg/kg), and *P* is the consumed portion (kg). This equation is presented as a simplified deterministic relationship. In probabilistic applications, *C* and *P* may be represented as distributions, yielding a corresponding distribution of *D*; units must remain consistent throughout the calculation.

Exposure estimates depend on representative sampling and analytical assumptions. Cross-contact can be highly heterogeneous, especially for discrete particles, so sampling should reflect expected contamination patterns and account for analytical and marker-conversion uncertainty.

### 7.2. Clinical Threshold Distributions, Reference Doses, and Action Levels

Oral food-challenge data can be used to construct population eliciting-dose distributions. ED01 and ED05 denote doses expected to elicit objective symptoms in 1% and 5% of the relevant food-allergic population, respectively [80,82]. These values describe population-level distributions rather than universally safe doses for every sensitized individual. Estimating these values depends on statistical modeling and data handling, the characteristics of the study population, and relevant cofactors [80,82].

Reference doses derived from these distributions provide health-based benchmarks for low, but not zero, residual risk and should not be interpreted as biological thresholds guaranteeing that reactions will or will not occur.

Once a health-based reference dose has been selected, a product-specific concentration-based action level can be derived using an explicit consumption assumption:

$$\mathrm{AL}=\frac{\mathrm{RfD}}{P}$$

where *AL* is the action level (mg/kg), *RfD* is the reference dose (mg allergenic-source protein), and *P* is the consumed portion (kg). This deterministic relationship assumes a defined portion size and consistent units; when *RfD* is expressed in milligrams of allergenic-source protein and *P* in kilograms of food, *AL* is expressed in mg/kg.

Because portion sizes vary, the same reference dose can produce different concentration-based action levels for different foods. Deterministic approaches tend to use a conservative portion size, while probabilistic approaches incorporate the distributions of concentration, intake amount, and clinical thresholds. The assumptions underlying consumption, treatment of non-detects, analytical and sampling uncertainty, and acceptable residual risk should therefore be explicitly stated.

### 7.3. Processing- and Matrix-Aware Risk Interpretation

Processing and food matrix effects complicate translation from measured concentration to clinical risk. No generally accepted factor converts changes in IgE binding, structural stability, or simulated gastrointestinal persistence into changes in human eliciting dose. Reference doses or clinical thresholds should therefore not be adjusted from any isolated molecular endpoint.

Reduced IgE recognition may indicate mechanistic change but not reduced clinical elicitation, while persistence of a molecular marker does not establish retained biological activity. Risk interpretation should therefore distinguish between molecular persistence and immune recognition from functional activity, exposure, and clinical outcomes. Claims of reduced risk after processing require evidence appropriate to support the inference being drawn.

Depending on the question at hand, a weight-of-evidence assessment may integrate molecular characterization with immunological, functional, and exposure data, as well as clinical evidence where justified and available. Hydrolyzed and fermented ingredients therefore require risk interpretation based on the biologically relevant species that remain, rather than on the disappearance of the intact parent protein.

### 7.4. Precautionary Allergen Labeling as Risk Management

Precautionary allergen labeling (PAL) belongs to risk management rather than hazard identification or analytical detection. Analytical evidence informs PAL through quantitative exposure assessment, but the detection of allergenic-source material alone does not determine whether PAL is warranted. Decisions should integrate exposure estimates with the applicable reference-dose framework, product-specific action levels, preventive-control performance, and documented sampling and analytical uncertainty [82,83,84].

The official FAO/WHO account of the 2026 Codex precautionary allergen labeling (PAL) guidance describes PAL as a risk-management tool rather than a substitute for allergen controls. It states that PAL should follow appropriate allergen-management measures and a scientific assessment of residual risk from unintended allergen presence [84]. Within this framework, reference doses provide health-based benchmarks, while product-specific action levels translate these benchmarks into concentration-based decision criteria using defined consumption assumptions [82,83,84].

The distinction among analytical measurand, exposure, biological activity, and clinical risk extends beyond the scope of any single regulatory framework. Codex guidance provides the current international risk-management context for translating exposure and reference-dose concepts into decisions. Table 5 summarizes the variables that should remain distinct.

### 7.5. Emerging Proteins and Risk-Assessment Priorities

Novel and emerging food sources, including edible insects and microalgae, may introduce insufficiently characterized proteins into novel food matrices, some of which share sequence or structural homology with known allergens. A study of crustacean-allergen detection methods also reported assay cross-reactivity with edible-insect samples [85]. In a separate primary study, sera from crustacean-allergic patients recognized house cricket and desert locust proteins, while sera from house dust mite–allergic patients bound proteins from these insects; these in vitro findings do not establish clinical cross-allergy after ingestion [86,87]. Sequence or structural similarity can contribute to hazard identification, but neither alone is sufficient to predict de novo sensitization, IgE-mediated functional activity, or clinical reactivity. A tiered, weight-of-evidence assessment should integrate molecular characterization across processing and gastrointestinal transformation, together with anticipated exposure and progressively stronger immunological, functional, and, where available, clinical evidence [10,72].

The same principle applies to proteins or processing strategies described as ‘hypoallergenic’. Reduced IgE binding is informative but is not equivalent to reduced effector-cell activity or a quantifiable increase in human eliciting dose; the evidentiary standard should match the claim.

**Figure 4:**
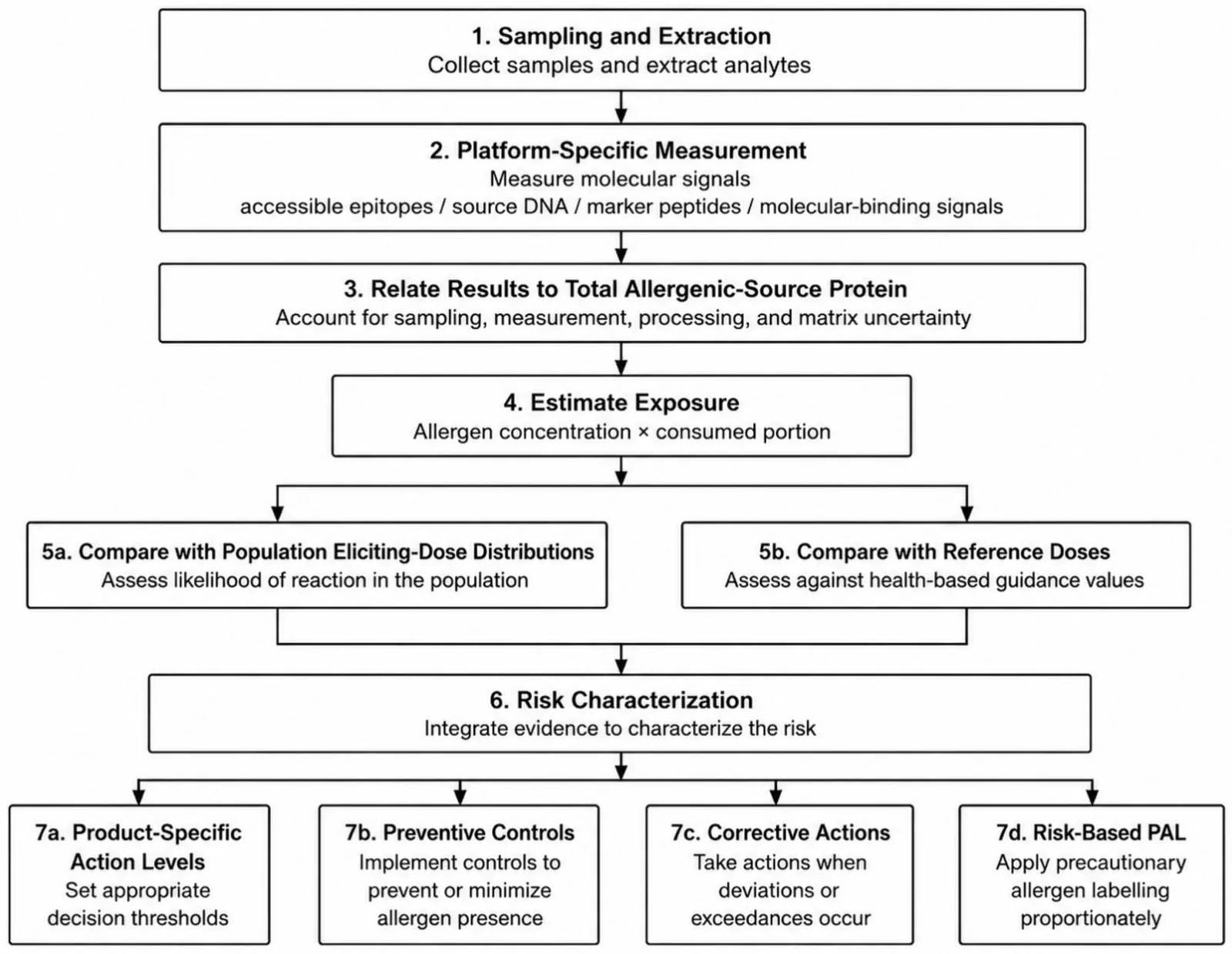
From molecular detection to risk-relevant interpretation. Sampling and extraction are followed by platform-specific measurement of accessible antibody-binding epitopes, source-specific DNA, selected peptide markers, or recognition-dependent signals. Where applicable, these measurands are related to total allergenic-source protein using validated analytical relationships while accounting for sampling, processing, matrix, calibration, and measurement uncertainty. Estimated exposure is derived from allergenic-source protein concentration and portion size and compared with population eliciting-dose distributions and reference doses. Product-specific action levels, preventive controls, corrective actions, and precautionary allergen labeling are then considered within an appropriate risk-management framework. Abbreviations: DNA, deoxyribonucleic acid; PAL, precautionary allergen labeling.

Further progress requires closer alignment among clinical threshold science, analytical metrology, molecular characterization, and exposure assessment. Priorities include greater harmonization of allergenic-source protein reporting and marker conversion, representative matrix-matched reference materials, and more robust interlaboratory characterization of analytical and sampling uncertainty. Greater consistency would improve traceability from molecular detection through exposure estimation to risk-management decisions. Figure 4 summarizes this progression.

## 8. Conclusions and Future Perspectives

IgE-mediated food allergenicity cannot be inferred from protein identity alone. Folding, disulfide connectivity, ligand binding, epitope organization, aggregation, and supramolecular assembly shape responses to processing, matrix association, and gastrointestinal transformation. Protein family membership can guide hypotheses about persistence and cross-recognition but does not predict sensitization, eliciting dose, or clinical severity.

Interactions between processing and the food matrix further diversify the molecular states of allergens, with impacts on structural organization, solubility, epitope accessibility, and digestive fate. These effects are context-dependent and cannot be inferred from processing intensity or composition alone. Gastrointestinal transit continues this process of molecular selection, generating persistent molecular species with variable IgE recognition and functional activity.

Analytical interpretation must remain measurand-specific. Since analytical platforms interrogate distinct molecular targets, their outputs are complementary but cannot be treated as interchangeable measures of allergen abundance, biological activity, exposure, or clinical risk. Reliable interpretation thus requires matrix-appropriate validation and explicit consideration of recovery, calibration, and measurement uncertainty.

A defensible risk framework links analytical results to total allergenic-source protein, consumed dose, eliciting-dose distributions, reference doses, and stated uncertainty. Reduced analytical recovery, altered IgE binding, or increased degradation should not be interpreted as lower clinical risk without evidence appropriate to that inference.

Several methodological priorities follow from this framework. Research studies should adopt harmonized processing and gastrointestinal digestion protocols, and apply structural, proteomic, IgE-binding, and effector-cell endpoints to the same realistically processed materials. Analytical laboratories require matrix-matched incurred reference materials, clearly characterized recovery and measurement uncertainty, and validated marker-to-total-protein relationships. Risk assessment should align analytical results with exposure and population threshold data while accounting for sampling uncertainty. For novel or modified proteins, evidence should progress from molecular characterization and exposure assessment to functional testing and, where justified, clinical evaluation rather than relying on sequence similarity or reduced IgE binding alone [6,9,11,75].

More physiologically representative systems, including organoids, co-culture models, and organ-on-a-chip platforms, may clarify epithelial and immune-interface processes not captured by static digestion models if their outputs are standardized against molecular and functional endpoints [88]. Computational approaches may help integrate molecular, analytical, exposure, and clinical threshold data, but they should complement rather than replace empirical validation, and should be tested against functional and, where available, clinical evidence. No single structural feature, processing response, digestion endpoint, or analytical signal establishes clinical risk; progress depends on connecting molecular state, measurand, biological activity, exposure, and clinical evidence while defining remaining uncertainty.

## Figures and Tables

**Table 1:** Major food allergen families, representative allergens, conserved structural motifs, and molecular determinants of allergen behavior.

Protein Family or Group	Representative Allergens	Conserved Structural Motif	Molecular Behavior and Representative Sources
Cupins	Ara h 1, Ara h 3; Gly m 5, Gly m 6	Conserved β-barrel cupin domains; oligomeric assemblies	Compact globular architecture, oligomerization, aggregation tendency, and context-dependent processing stability. Sources: peanut, soybean, tree nuts, and seeds [19,20,21].
2S albumins	Ara h 2, Ara h 6; Ses i 1; Ber e 1	Disulfide-rich α-helical seed-storage fold	In peanut Ara h 2/Ara h 6, disulfide-constrained structure is associated with proteolytic resistance and persistence of IgE-reactive digestion products. The listed sesame and Brazil-nut proteins are family examples, not directly tested in these peanut studies [26,27,28].
nsLTPs	Pru p 3; Ara h 9; Cor a 8	Four-helix bundle with four conserved disulfide bonds and a hydrophobic ligand-binding cavity	The family shares a ligand-binding fold; ligand-dependent changes in stability or IgE recognition have been studied in specific nsLTPs, including Pru p 3 and Len c 3. These results should not be generalized to every listed allergen or treated as proof of clinical cross-allergy [29,32,33].
PR-10 proteins	Ara h 8; Mal d 1; Cor a 1	Bet v 1-like fold with an internal cavity	Generally, heat- and digestion-labile and commonly associated with pollen–food cross-reactivity. Sources: fruits, nuts, and legumes [34].
Profilins	Bet v 2 homologues; Hev b 8	Conserved actin-binding fold	Broad IgE cross-recognition with generally limited thermal and digestive stability. Sources: diverse plant foods [34,38].
Wheat gluten proteins	ω-5 gliadin (Tri a 19); other gliadins and glutenins	Proline- and glutamine-rich gliadins; disulfide-linked glutenin polymers	Gliadins and disulfide-linked glutenin polymers contribute to gluten-network structure [22]. ω-5 gliadin has been implicated in wheat-dependent exercise-induced anaphylaxis [41]; processing-dependent analytical extractability requires separate validation.
Tropomyosins	Pen a 1; Pen m 1	Elongated α-helical coiled-coil structure	High sequence conservation and cross-recognition among invertebrates. Sources: crustaceans and mollusks [24].
Parvalbumins	Gad c 1; Cyp c 1	EF-hand calcium-binding motifs	Calcium binding can change parvalbumin conformation and IgE recognition in the tested fish proteins; the magnitude and direction are protein- and assay-dependent. Source: fish [35].
Milk proteins	Bos d 8 (caseins); Bos d 5 (β-lactoglobulin); Bos d 4 (α-lactalbumin)	Flexible phosphoprotein assemblies or compact globular whey-protein folds	Casein micellar organization, ligand binding, heat-induced aggregation, disulfide exchange, and persistence of linear epitopes. Source: cow’s milk [18,47].
Egg proteins	Gal d 1; Gal d 2; Gal d 3; Gal d 4	Kazal inhibitor, serpin, transferrin, and lysozyme folds	Markedly different thermal stability, aggregation behavior, and digestive persistence among individual allergens. Source: hen egg [23,43].

Abbreviations: nsLTPs, nonspecific lipid transfer proteins; PR-10, pathogenesis-related class 10.

**Table 2:** Major processing modalities and their context-dependent molecular consequences for food allergens.

Processing Modality	Dominant Molecular Events	Potential Reduction in Immunoreactivity	Potential Preservation or Increase in Immunoreactivity	Interpretive Requirement
Thermal processing	Unfolding, fragmentation, hydrolysis, aggregation, disulfide exchange, and Maillard reactions	Loss of conformational epitopes; extensive fragmentation	Exposure of linear epitopes; formation of multivalent aggregates; persistence of structurally stable regions	Interpret with solubility, extraction recovery, patient-serum IgE binding, and functional assay results; the cited general processing reviews do not establish a uniform clinical effect [6,7].
High-pressure processing	Volume-dependent conformational changes, altered hydration, oligomer dissociation, and aggregation with limited disruption of covalent bonds	Changes in recognition of native conformational epitopes under specified pressure–temperature conditions	Retention of disulfide-stabilized structures; exposure of buried regions; pressure-induced aggregation	Outcome depends on pressure level, holding time, temperature, pH, ionic strength, and food matrix [6,50,51].
Fermentation	Microbial proteolysis and acidification; other changes depend on the organism and substrate	Degradation of the parent protein may reduce some molecular readouts	IgE binding may persist despite degradation of the parent protein, as observed for β-lactoglobulin under the tested fermentation conditions	Microbial strain, substrate, fermentation time, pH, and proteolytic profile should be reported explicitly [6,40].
Enzymatic hydrolysis	Site-specific or broad proteolytic cleavage	Reduced abundance and multivalency of IgE-reactive molecular species after extensive hydrolysis	Residual IgE-reactive products may remain; retention of T-cell epitopes or disulfide-linked fragments requires separate evidence	Disappearance of intact protein is not evidence of complete immunological inactivation [6,40].
Glycation/Maillard chemistry	Carbonyl addition, advanced glycation-product formation, cross-linking, and generation of modified molecular surfaces	Chemical modification or masking of existing epitopes	Aggregation or altered recognition may occur in some systems; an increase in IgE binding cannot be inferred from studies reporting a decrease	Outcome depends strongly on sugar identity, water activity, temperature, pH, reaction time, protein structure, and matrix [6,7,49].
Oxidation and selected non-thermal chemical treatments	Treatment-dependent modification of susceptible protein groups; the chemical changes must be characterized for the specific system	Modification of susceptible residues, structural disruption, and reduced epitope recognition	Modified epitopes, aggregation, or altered protease accessibility may occur in tested systems; these are not established effects of every oxidative treatment	State the actual treatment and endpoint; seafood non-thermal processing evidence does not generalize to all chemical treatments [6,7,50].

Abbreviation: IgE, immunoglobulin E.

**Table 3:** Food matrix components and their effects on allergen structure, gastrointestinal fate, and analytical recovery.

Matrix Component	Representative Interactions	Structural or Processing Consequences	Gastrointestinal Consequences	Analytical Implications
Lipids	Hydrophobic binding, interfacial adsorption, ligand occupancy, and lipid-oxidation-derived adduct formation	Altered folding stability, aggregation behavior, thermal response, and epitope accessibility	May sequester or stabilize allergens, alter gastric release, or modify bile-mediated proteolysis	Lipid-rich matrices can affect extraction and target recovery; quantify these effects in the specific food and method rather than assuming a proportional change in marker peptides [9,10,12,13].
Carbohydrates and polysaccharides	Excluded-volume effects, viscosity changes, electrostatic association, physical entrapment, and covalent glycation	Altered hydration, molecular mobility, aggregation, Maillard chemistry, and processing-induced cross-linking	May alter enzyme diffusion, allergen release, and digestion kinetics	Entrapment and glycation can affect analytical recovery; claims about different water activities or immunoassay versus MS recovery require matrix-specific validation [11,12,13].
Polyphenols	Noncovalent binding through hydrogen bonding, hydrophobic and π–π interactions, and oxidative covalent coupling	Epitope masking, precipitation, altered surface properties, cross-linking, and modified protease accessibility	May alter solubility, protease access, and the profile of persistent digestion products	Can suppress extraction or antibody recognition; effects depend on polyphenol identity, stoichiometry, pH, oxidation state, and food matrix [12].
Mineral ions and salts	Coordination, electrostatic screening, ionic-strength effects, and ligand-dependent stabilization	Changes in conformation, micellar organization, aggregation, and stability of ligand-binding proteins	May stabilize or destabilize ligand-dependent molecular states and alter protease accessibility	Buffer and extraction conditions can affect measurements; the cited fish parvalbumin study directly addresses calcium-dependent IgE recognition, not cross-method assay comparability [35,59].
Other proteins and supramolecular networks	Protein–protein association, co-aggregation, disulfide exchange, Maillard cross-linking, and physical entrapment	Formation of mixed aggregates, dense protein networks, and poorly soluble complexes	Protein networks may alter release and digestion of allergens; epithelial accessibility requires a separately validated transport model.	Matrix-matched incurred materials can be used to assess extraction and marker recovery in processed foods; marker-to-total-protein conversion needs independent calibration [9,10,61].

Abbreviation: MS, mass spectrometry.

**Table 4:** Comparison of analytical platforms for detecting structurally dynamic food allergens.

Platform Category	Primary Measurand/Principle	Principal Strengths	Critical Limitations	Best-Fit Analytical Role
Antibody-based immunoassays, including ELISA, LFIA, and Western blot	Accessible antibody-binding epitopes on extracted protein species	Established and sensitive; ELISA supports routine quantitative analysis; LFIA enables rapid screening; Western blot provides molecular-size-resolved characterization of immunoreactive proteins or fragments	Epitope dependence, cross-reactivity, processing-induced epitope masking or modification, matrix interference, extraction losses, and assay-specific calibration. Denaturation, aggregation, glycation, oxidation, or fragmentation may alter antibody recognition without eliminating allergenic-source protein	Routine screening or quantification when matrix-specific recovery is validated; rapid on-site testing by LFIA; characterization of immunoreactive proteins or fragments by Western blot [9,10,58]
DNA-based amplification methods, including qPCR, dPCR, and LAMP	Source-specific DNA sequences detected or amplified enzymatically	High specificity for biological-source identification; useful when proteins are extensively modified or suitable antibodies are unavailable; dPCR supports low-copy quantification; LAMP enables rapid isothermal amplification	DNA is an indirect surrogate for allergenic-source protein. DNA and protein may persist, degrade, or be removed differently during processing; matrix inhibition, contamination, primer specificity, and low-DNA ingredients can affect interpretation	Source confirmation, trace screening, and complementary method validation; not a direct measure of allergenic-protein exposure [69,71,72,78,79]
Targeted LC–MS/MS	Selected proteotypic marker peptides generated after protein extraction and enzymatic digestion	High molecular specificity and potential multiplex analysis; isotope-labeled standards can aid quantification when recovery and calibration are validated	Sensitive to extraction and digestion recovery, peptide selection, modification, ion suppression, matrix interference, and marker-to-total-protein conversion	Quantitative confirmatory analysis and multiplex detection when marker peptides, recovery, digestion efficiency, calibration, and conversion factors have been validated [9,10,61,73]
LC–HRMS and discovery proteomics	Broad peptide or protein signatures, including fragments, sequence variants, PTMs, and processing-derived molecular species	Supports untargeted or semi-targeted molecular characterization, digestome mapping, modification analysis, and marker discovery	Requires instrumentation and bioinformatics; practical throughput and interlaboratory reproducibility depend on the validated workflow	Mechanistic characterization, marker discovery, and mapping of processing- or digestion-derived molecular species rather than routine release testing [9,10]
Biosensors and microfluidic platforms	Recognition-dependent optical, electrochemical, mechanical, or related instrumental signals	Potential for rapid, low-volume and portable testing; multiplex performance depends on device-specific validation	Matrix interference, surface fouling, nonspecific adsorption, sensor drift, device-specific calibration, limited harmonization, and reproducibility constraints; recognition elements may remain sensitive to processing-induced target remodeling	Rapid screening and process verification, with positive, borderline, or discordant results confirmed using validated laboratory methods [74,75]

Abbreviations: dPCR, digital polymerase chain reaction; ELISA, enzyme-linked immunosorbent assay; HRMS, high-resolution mass spectrometry; LAMP, loop-mediated isothermal amplification; LC–HRMS, liquid chromatography–high-resolution mass spectrometry; LC–MS/MS, liquid chromatography–tandem mass spectrometry; LFIA, lateral-flow immunoassay; PCR, polymerase chain reaction; PTMs, post-translational modifications; qPCR, quantitative polymerase chain reaction.

**Table 5:** Molecular evidence, decision variables, uncertainties, and interpretive roles in food allergen risk assessment.

Assessment Element	Molecular or Quantitative Evidence	Critical Uncertainty	Risk-Interpretation Role
Hazard identification	Allergen identity, protein family, sequence or structural homology, epitope information, abundance, and relevant molecular stability [19,34,70]	Incomplete allergen databases, isoform diversity, matrix context, uncertainty in clinical relevance of cross-recognition, and limited evidence for novel proteins	Prioritizes possible allergenic hazards for further assessment; molecular similarity alone cannot establish clinical reaction probability or severity [4,70].
Exposure assessment	Estimated concentration of total allergenic-source protein and an appropriate consumed portion; peptide-marker measurements require validated conversion to total protein [9,10,61,73].	Heterogeneous cross-contact, sampling design, extraction recovery, marker-to-total-protein conversion, measurement uncertainty, and portion-size distribution	Estimates ingested allergenic-source protein dose only when concentration and portion estimates are fit for purpose, enabling comparison with a selected reference dose [82,83,84].
Reference-dose comparison	Population eliciting-dose distributions and the selected health-based reference dose [80,82]	Censored challenge data, model choice, population representativeness, cofactors, and the accepted level of residual risk	Determines whether estimated exposure is consistent with the selected health-based benchmark and supports derivation of product-specific action levels [80,82,83]
Processing- and matrix-aware interpretation	Residual allergenic-source protein, persistent molecular species, peptide profiles, IgE-binding capacity, effector-cell activity, and gastrointestinal-transformation data [3,11,50]	No universal quantitative conversion from structural modification, in vitro immunoreactivity, or simulated digestion to human eliciting dose	Supports weight-of-evidence interpretation and prevents molecular modification or reduced assay response from being equated automatically with allergen elimination or reduced clinical risk [3,5,9,10]
Risk communication and controls	Reference dose, product-specific action level, exposure scenario, analytical uncertainty, contamination pattern, and effectiveness of preventive controls [80,82,83,84]	Sampling uncertainty, method limitations, jurisdictional requirements, communication wording, and consumer interpretation	Informs preventive controls and risk communication; PAL decisions must be checked against applicable guidance and verified residual exposure rather than inferred from the adoption announcement alone [82,83,84].
Novel proteins and hypoallergenic design	Sequence and structural similarity, protein abundance, processing behavior, gastrointestinal persistence, serum IgE recognition, and functional activity [4,8,70]	Sparse clinical data, potential novel epitopes, altered matrix exposure, limited post-market evidence, and uncertain population susceptibility	Proposes a tiered evidence-generation path; these molecular and analytical indicators do not independently establish reduced clinical allergenicity [10,70].

Abbreviation: IgE, immunoglobulin E.

## Data Availability

No new data were created or analyzed in this study. Data sharing is not applicable to this article.
